# Evaluation of Fourier Transform Infrared Spectroscopy as a First-Line Typing Tool for the Identification of Extended-Spectrum β-Lactamase-Producing *Klebsiella pneumoniae* Outbreaks in the Hospital Setting

**DOI:** 10.3389/fmicb.2022.897161

**Published:** 2022-06-09

**Authors:** Jun Hao Wang-Wang, Antoni E. Bordoy, Elisa Martró, María Dolores Quesada, María Pérez-Vázquez, Mercedes Guerrero-Murillo, Andrea Tiburcio, Marina Navarro, Laia Castellà, Nieves Sopena, Irma Casas, Verónica Saludes, Montserrat Giménez, Pere-Joan Cardona

**Affiliations:** ^1^Microbiology Department, Laboratori Clínic Metropolitana Nord, Germans Trias i Pujol University Hospital, Badalona, Spain; ^2^Genetics and Microbiology Department, Universitat Autònoma de Barcelona, Bellaterra, Spain; ^3^Germans Trias i Pujol Research Institute (IGTP), Badalona, Spain; ^4^Centro de Investigación Biomédica en Red en Epidemiología y Salud Pública (CIBERESP), Instituto de Salud Carlos III (ISCIII), Madrid, Spain; ^5^Reference and Research Laboratory for Antibiotic Resistance and Health Care Infections, National Centre for Microbiology, Instituto de Salud Carlos III (ISCIII), Madrid, Spain; ^6^Centro de Investigación Biomédica en Red en Enfermedades Infecciosas (CIBERINFEC), Instituto de Salud Carlos III (ISCIII), Madrid, Spain; ^7^Clinical Genomics Research Unit, Germans Trias i Pujol Research Institute (IGTP), Can Ruti Campus, Badalona, Spain; ^8^Clinical Genomics Unit, Clinical Genetics Service, Laboratori Clínic Metropolitana Nord, Germans Trias i Pujol University Hospital, Can Ruti Campus, Badalona, Spain; ^9^Enfermería Control de Infección, Dirección Enfermería, Germans Trias i Pujol University Hospital, Badalona, Spain; ^10^Infectious Diseases Department, Germans Trias i Pujol University Hospital, Badalona, Spain; ^11^Preventive Medicine Department, Germans Trias i Pujol University Hospital, Badalona, Spain; ^12^Centro de Investigación Biomédica en Red en Enfermedades Respiratorias (CIBERES), Instituto de Salud Carlos III (ISCIII), Madrid, Spain

**Keywords:** cluster analysis, Fourier transform infrared spectroscopy, FTIR, *Klebsiella pneumoniae*, outbreak, whole-genome sequencing, conventional epidemiology, nosocomial infection

## Abstract

Early detection of pathogen cross-transmission events and environmental reservoirs is needed to control derived nosocomial outbreaks. Whole-genome sequencing (WGS) is considered the gold standard for outbreak confirmation, but, in most cases, it is time-consuming and has elevated costs. Consequently, the timely incorporation of WGS results to conventional epidemiology (CE) investigations for rapid outbreak detection is scarce. Fourier transform infrared spectroscopy (FTIR) is a rapid technique that establishes similarity among bacteria based on the comparison of infrared light absorption patterns of bacterial polysaccharides and has been used as a typing tool in recent studies. The aim of the present study was to evaluate the performance of the FTIR as a first-line typing tool for the identification of extended-spectrum β-lactamase-producing *Klebsiella pneumoniae* (ESBL-Kp) outbreaks in the hospital setting in comparison with CE investigations using WGS as the gold standard method. Sixty-three isolates of ESBL-Kp collected from 2018 to 2021 and classified according to CE were typed by both FTIR and WGS. Concordance was measured using the Adjusted Rand index (AR) and the Adjusted Wallace coefficient (AW) for both CE and FTIR clustering considering WGS as the reference method. Both AR and AW were significantly higher for FTIR clustering than CE clustering (0.475 vs. 0.134, *p* = 0.01, and 0.521 vs. 0.134, *p* = 0.009, respectively). Accordingly, FTIR inferred more true clustering relationships than CE (38/42 vs. 24/42, *p* = 0.001). However, a similar proportion of genomic singletons was detected by both FTIR and CE (13/21 vs. 12/21, *p* = 1). This study demonstrates the utility of the FTIR method as a quick, low-cost, first-line tool for the detection of ESBL-Kp outbreaks, while WGS analyses are being performed for outbreak confirmation and isolate characterization. Thus, clinical microbiology laboratories would benefit from integrating the FTIR method into CE investigations for infection control measures in the hospital setting.

## Introduction

Nosocomial infections cause a high impact on the healthcare system due to an increase in antimicrobials use, patient morbidity and mortality, and hospitalization length. Nosocomial infection prevalence has ranged from 5% to 10% in recent years (World Health Organization, 2016, 2021). It is well established that early detection of pathogen cross-transmission events along with a quick implementation of correct infection control measures is required to prevent the spread of nosocomial infections (Brouqui et al., 2017; Haque et al., 2018). The bacterial species most frequently involved in nosocomial infections are *Enterobacter* spp., *Staphylococcus* spp., *Klebsiella* spp., *Acinetobacter* spp., *Pseudomonas* spp., and *Enterococcus* spp., which are also known as the ESKAPE pathogens and usually exhibit multidrug resistance and virulence (Santajit and Indrawattana, 2016; Antimicrobial Resistance Collaborators, 2022). Among them, *Klebsiella pneumoniae* is one of the microorganisms with the highest burden in healthcare settings (Santajit and Indrawattana, 2016). Specifically, in 2020, the reported rate of ESBL-Kp in Spain was 26.8% (European Centre for Disease Prevention and Control, 2020) and it was 21.5% in our hospital.

For successful nosocomial outbreak control, it is necessary to identify as soon as possible any potential transmission routes and any likely associated environmental reservoirs through conventional epidemiology (CE) investigations that must be confirmed later by bacterial typing. Pulsed-field gel electrophoresis (PFGE) has been widely used as the gold standard molecular method for outbreak identification based on bacterial typing (Peacock et al., 2002). However, over the last decade, whole-genome sequencing (WGS) has become the gold standard method due to its higher discriminatory power and increased accessibility (Bertelli and Greub, 2013; European Centre for Disease Prevention and Control, 2018). The potential of WGS for establishing precise relationships between bacterial isolates makes this technique the ideal tool for outbreak investigation in scenarios where other routinely used methods lack sensitivity (Peacock et al., 2018; European Centre for Disease Prevention and Control, 2019). Nevertheless, WGS-based laboratory methods and bioinformatics analyses require skilled staff and are laborious and time-consuming. Besides, bioinformatics analyses of WGS data require large computational resources to be processed, and the obtained results often require expert interpretation. Thus, WGS is difficult to implement in the routine clinical practice in most clinical microbiology laboratories. Therefore, the implementation of quicker and easier to use approaches for bacterial typing could facilitate the task of outbreak identification by CE teams in the hospital setting.

Fourier transform infrared (FTIR) spectroscopy is a phenotypic technique introduced in the 1950s based on the comparison of infrared light absorption patterns after the application of the Fourier transform (Naumann et al., 1991; Griffiths and de Haseth, 2007). Such patterns vary according to the functional groups present in biomolecules such as carbohydrates, lipids, and proteins. The polysaccharide region (1,200–900 cm^−1^) contributes more significantly than others to the spectral differences observed between strains from the same species (Helm et al., 1991; Naumann et al., 1991). Based on the polysaccharide region, recent studies have demonstrated that the most interesting wavelength of the infrared spectrum for bacterial typing is 1,300–800 cm^−1^ (Martak et al., 2019; Vogt et al., 2019). FTIR spectroscopy is used by the IR Biotyper system (Bruker GmbH, Leipzig, Germany), which offers a rapid and low-cost typing technique (both sample processing and amount and number of reagents are minimal) with a short turn-around time (3–4 h). Similarity between transformed spectra is established by multivariate data analysis of spectral peaks, and relationships between isolates can then be identified (Quintelas et al., 2018; Burckhardt et al., 2019; Martak et al., 2019; Novais et al., 2019; Rakovitsky et al., 2020). Thus, FTIR could be an alternative method for the quick identification of nosocomial outbreaks. However, the few studies performed so far regarding the utility of FTIR toward ESBL-Kp outbreak detection have focused on the comparison of FTIR typing results to genomic analysis, but none of them have compared FTIR to CE, although CE constitutes the most frequently used outbreak investigation tool in many healthcare settings. The aim of the present study was to evaluate the performance of the IR Biotyper as a first-line bacterial typing tool for the identification of ESBL-Kp outbreaks in the hospital setting in comparison with CE investigations using WGS as the gold standard method.

## Materials and Methods

### Study Design

A retrospective and cross-sectional study was performed in a tertiary care hospital. This center is the general hospital for 200,000 inhabitants and the reference hospital for 1,200,000 inhabitants of the Northern Metropolitan Area of Barcelona (Catalonia, Spain).

The study dataset comprised 63 ESBL-Kp isolates collected from patients admitted to the reference hospital (*n* = 61) and from the environment (*n* = 2) from July 2018 to October, 2021. Among them, 40 isolates belonged to epidemiological outbreaks identified by CE during the study period by the hospital infection prevention and control team. The CE criterion used in the present study to declare a nosocomial outbreak (epidemiological cluster) was the detection of two or more nosocomial ESBL-Kp infections (detected after the first 48 h of hospital admission which were not present at the time of admission; Horan et al., 2008) in the same hospital unit/ward in less than a month (Centers for Disease Control and Prevention, 2012). Additionally, in critical units (cardiac care unit, intensive care unit, neonatal intensive care unit, and both SARS-CoV-2 intensive care units) nosocomial ESBL-Kp colonization cases were also considered for outbreak declaration. The date of outbreak closure was set when no ESBL-Kp isolates were identified in the same hospital unit/ward for 3 months since the last identification. The remaining 23 isolates were randomly selected among cases that did not meet the nosocomial outbreak criteria and therefore were considered epidemiological singletons.

ESBL-Kp isolates were pseudonymized using a consecutive number in order to maintain patient confidentiality. All ESBL-Kp isolates were obtained for routine clinical diagnosis purpose and no additional sampling was required; therefore, informed consent was waived.

### Routine Microbiological Diagnostics and Antimicrobial Susceptibility Testing

All isolates were firstly identified at the species level by matrix-assisted laser desorption ionization-time of flight mass spectrometry (MALDI-TOF MS, Bruker Daltonik GmbH, Bremen, Germany). Antibiotic susceptibility was interpreted following European Committee on Antimicrobial Susceptibility Testing (EUCAST) criteria (The European Committee on Antimicrobial Susceptibility Testing, 2021). All isolates were subsequently conserved in storage media at −80°C until further analyses.

### Sample Preparation for FTIR Analysis

All ESBL-Kp isolates were cultured in BD MacConkey II agar medium (Becton Dickinson GmbH, Heidelberg, Germany) for 24 h at 37°C. Then, all bacterial isolates were subcultured in Mueller-Hinton agar medium from a single colony (Becton Dickinson GmbH, Heidelberg, Germany) for another 24 h at 37°C before testing. For bacterial suspension preparation, the modified H_2_O-EtOH protocol was used (Hu et al., 2021); a loopful of bacterial cells was suspended in 50 μl of deionized water in a 1.5-ml suspension vial with metal beads (Bruker GmbH, Leipzig, Germany) and homogenized through vortex. Then, 50 μl of 70% (v/v) ethanol was added in each vial and suspensions were homogenized again. Subsequently, 15 μl of each suspension vial was placed in quadruplicate on a 96-spot silicon plate (Bruker GmbH, Leipzig, Germany). As quality control, 12 μl of two infrared test standards (IRTS 1 and IRTS 2, each standard containing an *Escherichia coli* strain with a specific spectrum) was placed in duplicate on the same silicon plate. The plate was dried for 30 min at 37°C before introducing it into the Biotyper system.

### Spectrum Analysis of FTIR Data

For FTIR analysis, manufacturer’s recommendations were followed. Briefly, all samples were tested in quadruplicate using the default analysis settings (wavelength region 1,300–800 cm^−1^) and OPUS 8.2.28 software (Bruker GmbH, Leipzig, Germany). Samples that did not meet the quality criteria were excluded. The OPUS software generates an average spectrum using the four obtained spectra of each replicate for each sample. It then analyzes the similarity between the average spectra from different samples and generates a dendrogram based on hierarchical cluster analysis (HCA) using Euclidian metric distance and average linkage method. The FTIR software suggests a cutoff value for clustering based on Simpson’s Index of Diversity (Hunter and Gaston, 1988). For *K. pneumoniae*, manufacturer recommends using a cutoff ranging from 0.20 to 0.25, with an initial validation to determine the best value using a molecular method as gold standard. Accordingly, a subset of the study isolates comprising the first 17 isolates with available WGS results (12 forming a genomic cluster plus five singletons) was used to determine an initial cutoff. The obtained cutoff was then validated using all 63 isolates by maximizing the overall congruence between FTIR and WGS results. When two or more isolates had a FTIR spectrum distance less or equal to the designated cutoff value, they were considered to belong to the same FTIR cluster, and isolates that did not meet this criterion were considered FTIR singletons.

### Sample Preparation for WGS Analysis

All ESBL-Kp samples were cultured in Mueller–Hinton agar (Becton Dickinson GmbH, Heidelberg, Germany) overnight at 37°C. Then, a single colony was subcultured in thioglycolate broth (bioMérieux SA, Marcy-l’Étoile, France) overnight at 37°C with agitation. DNA was extracted from 1.5 ml of grown thioglycolate broth using the DNeasy Ultra Clean™ Microbial kit (Qiagen GmbH, Hilden, Germany) following manufacture’s recommendations. Nextera XT DNA Library Preparation Kit (Illumina Inc., San Diego, United States) was used for dual-indexed library preparation according to the manufacturer’s instructions, and paired-end sequencing was performed using the Illumina MiSeq platform (Illumina Inc., San Diego, United States).

### Bioinformatic Analyses of WGS Data

For WGS clustering analysis of ESBL-Kp isolates, two approaches were combined to increase the discriminatory power of WGS analysis as previously described (Raro et al., 2020): (i) core genome multilocus sequence typing (cgMLST) of 2,358 chromosomal target genes and (ii) phylogenetic analysis of core-genome single nucleotide polymorphisms (cgSNP). A genomic cluster of ESBL-Kp isolates by WGS was defined when the following two conditions were met based on an internal validation according to recorded epidemiological data: (i) ≥2 cases of the same sequence type (ST) with a genetic distance of ≤15 alleles in the cgMLST analysis and (ii) a common monophyletic origin in the phylogenetic tree supported by a bootstrap value ≥70% with a genetic distance of ≤17 SNP provided by cgSNP analysis. Isolates that did not meet these criteria were classified as genomic singletons. Each genomic cluster and singleton were considered a different ESBL-Kp introduction event into the hospital.

cgMLST analysis was performed with Ridom SeqSphere+ software 5.1.0 (Ridom GmbH, Münster, Germany; Jünemann et al., 2013) using *assembly de novo* settings with SPAdes 3.11.1 (Bankevich et al., 2012) and the parameter “pairwise ignoring missing values.” From this analysis, ST data were obtained, and a minimum spanning tree was built to visualize allele distance between isolates.

Prokka v.1.14 (Seemann, 2014) was used to annotate *de novo* assemblies, and these annotated assemblies were used as input for Roary v3.13.0 (Page et al., 2015). An alignment of 3,524 core genes (present in >99% of isolates) comprising 3,481,593 bp was generated. Variable positions of the alignment were extracted (78,071 SNPs), and a maximum-likelihood phylogenetic tree of SNPs was constructed using RAxML v7.0.4 (Stamatakis, 2006) with a general time-reversible (GTR) model and a gamma correction for site rate variation. Node support was assessed through bootstrapping with 100 replicates.

Additionally, resistance analysis was performed using KmerResistance 2.2 online tool with default parameters (Clausen et al., 2016, 2018; Center for Genomic Epidemiology, 2021). Those genes with “template_ID” lower than 98%, “template_coverage” lower than 100% and a “depth” that did not reach 40X were filtered out.

### Concordance Between Clustering Methodologies

To assess the concordance of the clustering results obtained by both the CE investigations and FTIR vs. WGS as the reference method, the Adjusted Rand index (AR) and the Adjusted Wallace coefficient (AW) were calculated with 95% CI using an online tool (Comparing Partitions, 2011). AR compares the overall congruence between two typing methods (Santos and Embrechts, 2009), while AW compares the agreement of two typing methods considering one of them as the reference method (Severiano et al., 2011). AR and AW values may range from 0 to 1, where 0 means agreement expected by chance and 1 means perfect correlation between both methods (Severiano et al., 2011; Hoffman et al., 2015). Additionally, *p* values comparing the AW and AR values for (i) CE vs. WGS clustering and (ii) FTIR vs. WGS clustering were obtained according to the jackknife pseudo-values resampling method (Comparing Partitions, 2011).

Besides, the proportion of isolates correctly clustered by either FTIR or CE was calculated using WGS as the reference method. Proportions were compared using the Fisher’s exact test. Statistical significance was set at *p* < 0.05.

## Results

### Conventional Epidemiology Identified Eight Outbreaks

CE investigations detected eight nosocomial outbreaks over the study period, which accounted for 40 out of 63 studied isolates. Specifically, the outbreaks were located at the CCU (outbreak A, *n* = 4), the OW (outbreak B, *n* = 2), the NPHD (outbreak C, *n* = 3; and outbreak H, *n* = 4), the ICU (outbreak D, *n* = 9; and outbreak E, *n* = 8), the SICU 1 (outbreak F, *n* = 4), and the SICU 2 (outbreak G, *n* = 6). According to the used CE criterion, the remaining 23 isolates presented no evident epidemiological links among them or the isolates related to outbreaks A-H. Distribution for all isolates according to source and ward where they were detected can be found in Supplementary Table 1; Supplementary Text 1.

### WGS Detected 32 Different Introductions of ESBL-Kp

**Figure 1:**
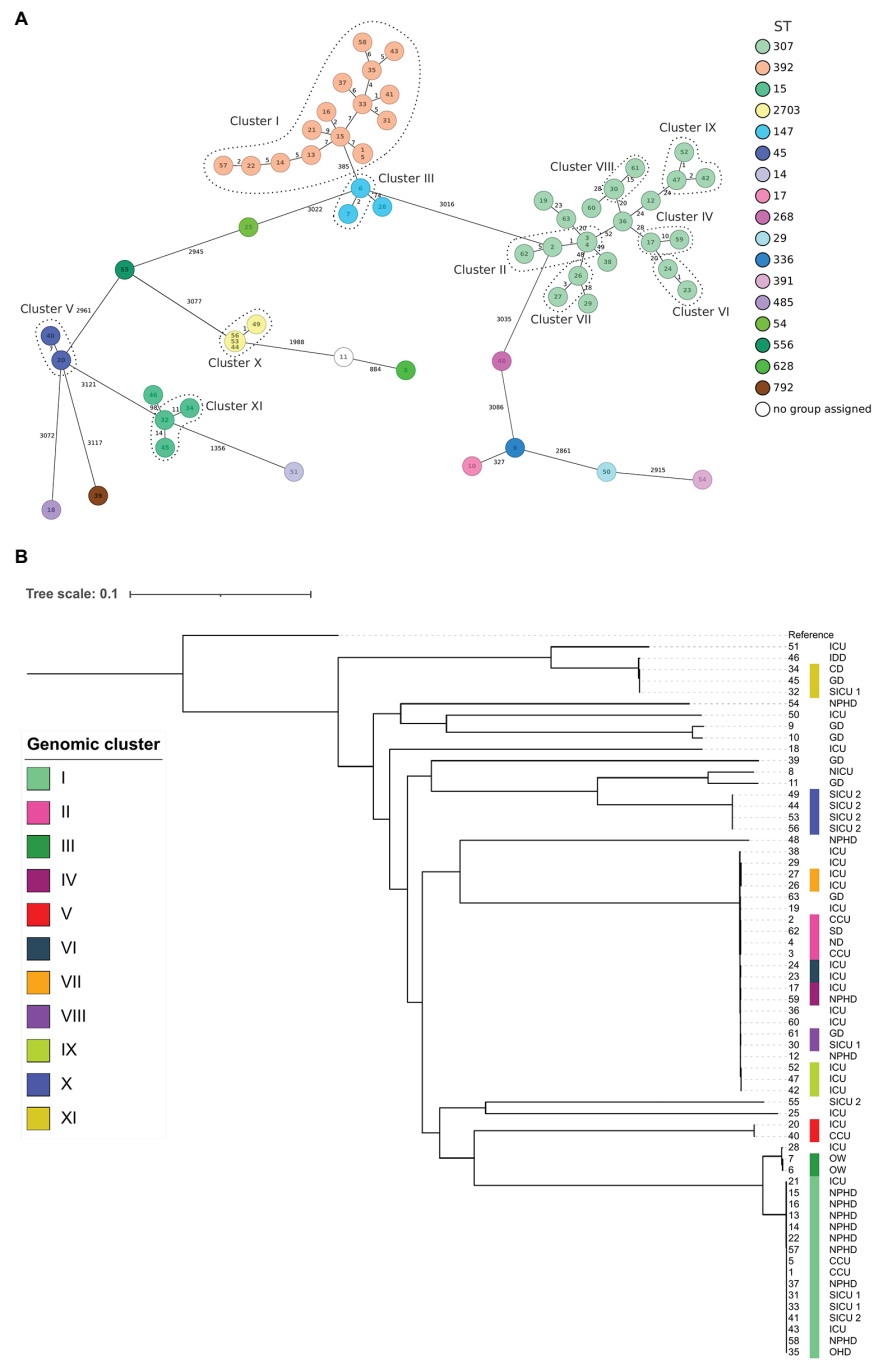
**(A)** Minimum spanning tree of extended-spectrum β-lactamase-producing *Klebsiella pneumoniae* (ESBL-Kp) isolates (*N* = 63) showing identified clusters (roman numerals and encircled dotted lines) and allele distance between strains based on cgMLST analysis of 2,358 genes. Each sequence type (ST) assigned is indicated with a different color. Branch lengths are not to scale. **(B)** Maximum-likelihood phylogenetic tree obtained from cgSNP analysis of ESBL-Kp isolates showing identified clusters (roman numerals and color legend). Hospital locations are also indicated (CCU, cardiac care unit; CD, cardiology department; FIGURE 1GD, geriatric department; ICU, intensive care unit; IDD, infectious disease department; ND, neurology department; NICU, neonatal intensive care unit; NPHD, nephrology department; OHD, oncohematology department; OW, obstetric ward; SD, surgery department; SICU 1, SARS-CoV-2 intensive care unit 1; and SICU 2, SARS-CoV-2 intensive care unit 2). Reference: NTUH-K2044 (accession no. NC_012731.1). The tree scale represents the number of substitutions per variable site. All nodes corresponding to each individual genomic cluster were supported with bootstrap values ≥70%.

The cgMLST allele distance among the 63 isolates is depicted in a minimum spanning tree shown in Figure 1A. Additionally, the phylogenetic tree of a cgSNP alignment of 78,071 high-quality sites is shown in Figure 1B. In both Figure 1 panels, the clusters that meet the combined cgMLST and cgSNP criteria are identified.

**Figure 2:**
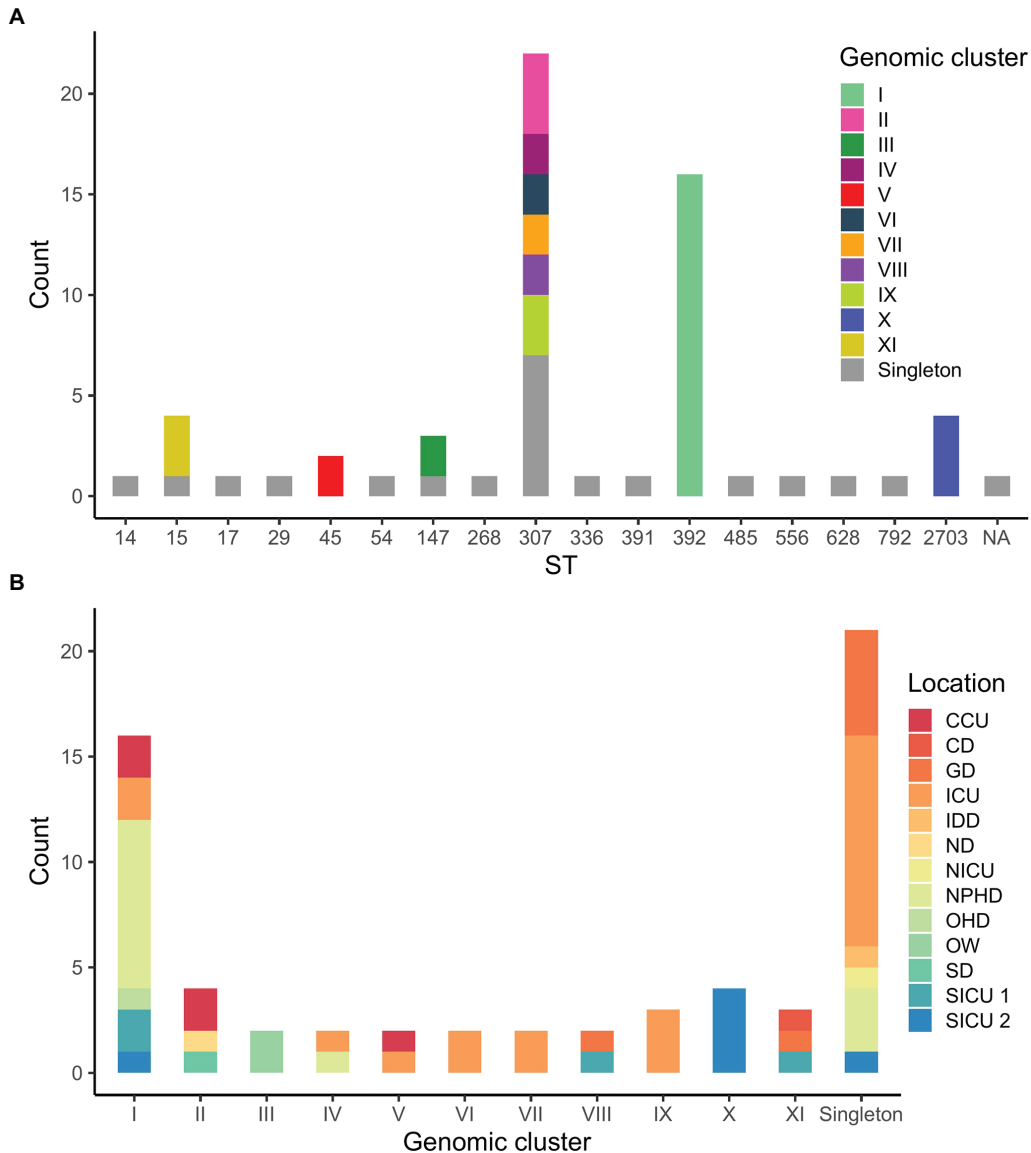
**(A)** Distribution of isolates according to their sequence type (ST). Genomic clusters of isolates for each ST are indicated in colors. NA, not assigned. **(B)** Distribution of isolates according to WGS clustering results. Hospital locations affected by each genomic clustering category are indicated in colors.

According to WGS clustering results, sequenced isolates represented at least 32 different introductions of ESBL-Kp into the hospital over the study period; 11 different clusters (namely I–XI, 42 isolates), plus 21 singletons were identified (Figure 1; Supplementary Table 1). WGS analysis confirmed two monoclonal outbreaks (B and C) and six polyclonal outbreaks (A, D, E, F, G, and H) caused by 10 different genomic clusters (I–IV and VI–XI) plus nine singletons (Table 1). The isolates were assigned to 17 different ST by cgMLST; among them, 41 out of 63 isolates (65.1%) belonged to high-risk clones ST147, ST307, and ST392, being ST307 the most prevalent one (22/63, 34.9%; Figures 1A, 2A). Genomic clusters I, III, V, X, and XI belonged to different STs, but genomic clusters II, IV, VI–IX belonged to ST307. Mean allele distances between isolates of a genomic cluster ranged from 1 to 9.5 (Supplementary Text 2). Six out of the 11 genomic clusters included isolates from more than one hospital location over the study period (Figure 2B). Cluster I isolates were the most spread in the hospital, affecting up to six different wards/units. The analysis of antibiotic resistance from WGS data confirmed the presence of at least one ESBL-resistance gene in all cases, being CTX-M-15 the most frequently detected ESBL, present in 95% (60/63) of the isolates (data not shown).

**Table 1:** Contingency table comparing epidemiological clustering (A–H) vs. WGS clustering (I–XI) as the reference method.

		WGS clustering												
		I	II	III	IV	V	VI	VII	VIII	IX	X	XI	S	Total
Epidemiological clustering	A	2	2											4
	B			2										2
	C	3												3
	D	1			1		2	2					3	9
	E	1								3			4	8
	F	2							1			1		4
	G	1									4		1	6
	H	2			1								1	4
	S	4	2			2			1			2	12	23
	Total	16	4	2	2	2	2	2	2	3	4	3	21	63

S, Singleton.

As for the comparison between WGS and CE results, outbreaks detected by CE included two or more isolates that were confirmed by WGS to belong to the same genomic cluster in 24 out of 42 (57.1%) cases. Moreover, 11 out of 23 (47.8%) isolates not identified as belonging to any outbreak according to CE formed or were part of five out of the six genomic clusters detected in more than one hospital location (I, II, V, VIII, and XI; Figure 2B). Finally, CE correctly identified 12 out of 21 (57.1%) genomic singletons (Table 1).

### FTIR Is a Useful First-Line Tool for Detection of ESBL-Kp Outbreaks

**Figure 3:**
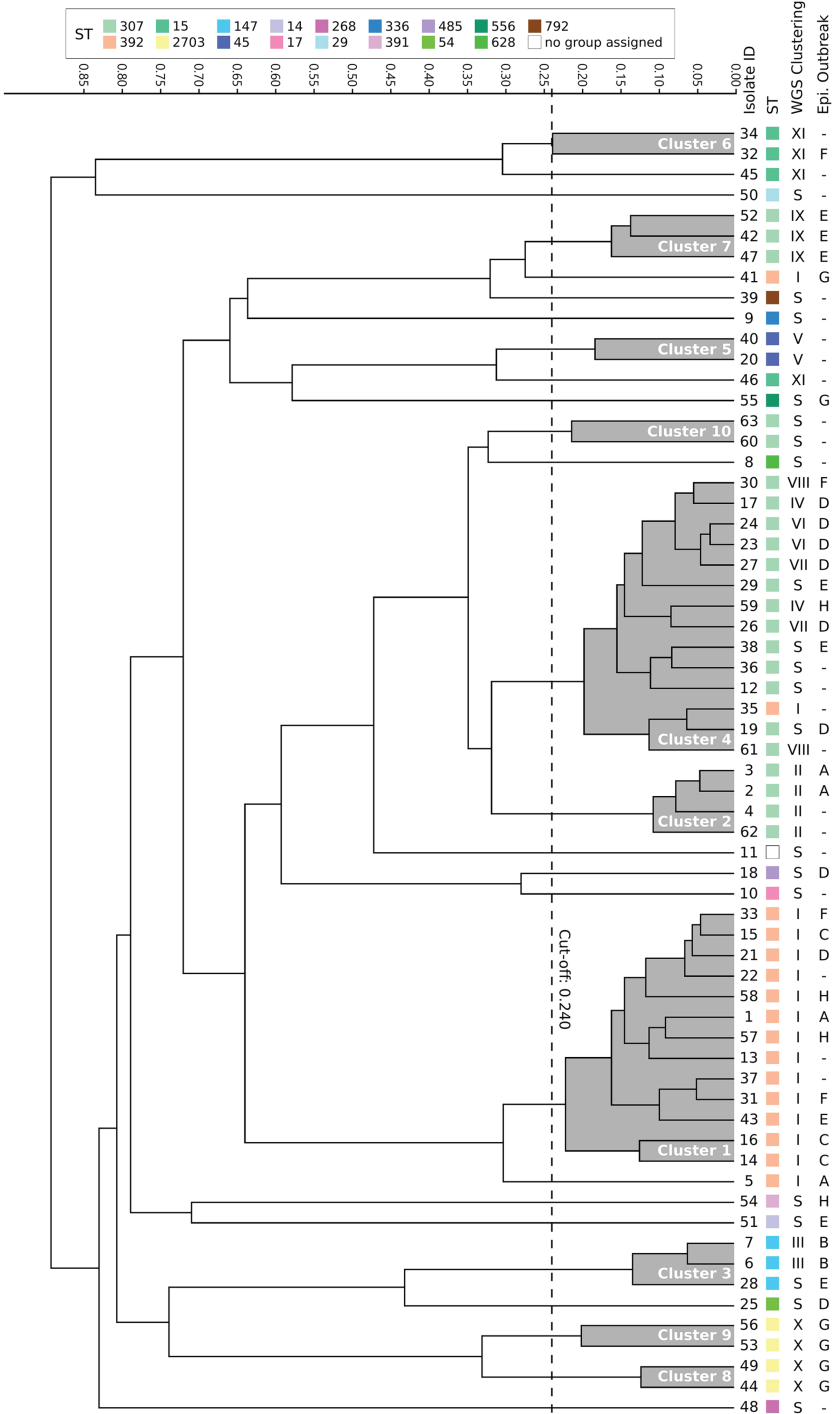
Dendrogram obtained by clustering the Fourier transform infrared spectroscopy (FTIR) spectra of 63 extended-spectrum β-lactamase-producing *K. pneumoniae* isolates. The vertical dashed line indicates the used cutoff value (0.240). Resulting FTIR clusters (1–10) are shadowed in gray. Corresponding sequence type (ST), WGS cluster, and epidemiological outbreak are indicated for each isolate. S, singleton.

For FTIR clustering, an optimal cutoff of 0.240 was obtained after an initial validation (*n* = 17) which coincided with the best cutoff value for the whole study dataset (*N* = 63; Supplementary Figure 1). Among all 63 ESBL-Kp isolates tested, FTIR detected 10 clusters (namely 1–10, including 47 isolates) and 16 singletons. The two largest clusters were cluster 4 (*n* = 14, 22.2%) and cluster 1 (*n* = 13, 20.6%; Figure 3; Table 2). Regarding the isolates initially classified into eight outbreaks by CE (*n* = 40), FTIR classified two outbreaks as monoclonal (B and C) and six as polyclonal (A, D, E, F, G, and H), similarly to WGS results (Supplementary Table 1). However, FTIR detected less ESBL-Kp clusters and singletons than WGS (8 vs. 10 and 7 vs. 9, respectively; Figure 3; Supplementary Table 1). Additionally, and in consonance with WGS, FTIR detected six clusters (1, 2, 4, 5, 6, and 10) affecting more than one hospital location. However, four (1, 4, 6, and 10) out of these six clusters were not fully concordant with WGS clustering (Table 2; Supplementary Text 3). In fact, when considering all studied isolates (*N* = 63), the concordance between FTIR and WGS was as follows: FTIR grouped in the same cluster singletons or isolates of different genomic clusters in three occasions (clusters 3, 4, and 10), a perfect agreement was observed between FTIR and genomic clusters in three cases (cluster 2/II, 5/V, and 7/IX), and finally, WGS grouped in the same genomic cluster isolates of different FTIR clusters in three instances (clusters I, X, and XI; Figure 3; Table 2). All this translates into an AR and AW values of 0.475 (95% CI, 0.262–0.691) and 0.521 (95% CI, 0.377–0.665), respectively, for FTIR clustering considering WGS as the reference method.

**Table 2:** Contingency table comparing FTIR clustering (1–10) vs. WGS clustering (I–XI) as the reference method.

		WGS clustering												
		I	II	III	IV	V	VI	VII	VIII	IX	X	XI	S	Total
FTIR clustering	1	13												13
	2		4											4
	3			2									1	3
	4	1			2		2	2	2				5	14
	5					2								2
	6											2		2
	7									3				3
	8										2			2
	9										2			2
	10												2	2
	S	2										1	13	16
	Total	16	4	2	2	2	2	2	2	3	4	3	21	63

S, Singleton.

In order to assess the value of FTIR over CE investigations in the hospital setting, AR and AW values were also calculated for CE clustering considering WGS as the reference method: AR was 0.134 (95% CI, 0.000–0.278) and AW was 0.134 (95% CI, 0.000–0.281). Both AR and AW were significantly higher for FTIR clustering than for CE clustering (0.475 vs. 0.134, *p* = 0.01, and 0.521 vs. 0.134, *p* = 0.009, respectively). To further evaluate the benefit of enhancing CE investigations using FTIR, we analyzed which of those isolates that were part of a genomic cluster (*n* = 42) were grouped by either FTIR or CE. FTIR clusters included two or more isolates that WGS confirmed to belong to the same genomic cluster in 38 out of 42 (90.5%) cases. Besides, 14 of 23 (45.7%) isolates not identified as belonging to any outbreak according to CE investigations were grouped into FTIR clusters detected in more than one hospital location (1, 2, 4, 5, 6, and 10), whereas FTIR correctly identified 13 out of 21 (61.9%) genomic singletons (Table 2). Therefore, FTIR significantly inferred more true genomic relationships than CE (38/42 vs. 24/42, *p* = 0.001). However, a similar proportion of genomic singletons was detected by both FTIR and CE (13/21 vs. 12/21, *p* = 1). Importantly, FTIR was able to detect a genomic relationship for isolates belonging to genomic clusters IV, V, VIII, and XI, which could be classified as “hidden outbreaks” because CE research was unable to establish any epidemiological link between them.

## Discussion

This study demonstrates the utility of the FTIR method as a rapid first-line laboratory tool for ESBL-Kp outbreak detection, while confirmatory results are being generated by WGS. FTIR inferred more true genomic relationships than CE. Moreover, FTIR detected four hidden outbreaks identified by WGS. Thus, FTIR adds valuable information that can be incorporated into CE investigations and does so in a timely manner.

A great performance of the FTIR method regarding the investigation of bacterial clusters has been suggested by a few studies performed so far. These evaluations have been carried out for *Klebsiella pneumoniae*, *Pseudomonas aeruginosa*, *Enterobacter cloacae*, and *Acinetobacter baumannii* (Dinkelacker et al., 2018, Martak et al., 2019; Vogt et al., 2019; Rakovitsky et al., 2020; Hu et al., 2021, Lombardo et al., 2021). Some of the previous studies employ a workflow different from the commercial one using the attenuated total reflection mode accessory of the FTIR instrument (Silva et al., 2020; Novais et al., 2022). However, although some of those studies use WGS-based analyses, most of them rely on PFGE and MLST as the reference molecular method and their isolates are often closely related, belonging to just one or a reduced number of outbreaks. Additionally, none of the previous studies compares FTIR vs. CE, being CE the most frequently used outbreak investigation tool in the healthcare setting. In the present study, a lower concordance between FTIR and WGS clustering methods (AR = 0.475, AW = 0.521) was obtained for *K. pneumoniae* isolates than in the previously published studies where AR ranged from 0.743 to 1.0 and AW ranged from 0.708 to 1.0 (Dinkelacker et al., 2018; Martak et al., 2019; Rakovitsky et al., 2020; Hu et al., 2021). Two methodological strengths of the present compared to previous studies may explain the lower concordance results observed here. Firstly, we analyzed isolates collected from more than one hospital location, from different patients and over a long period of time (3 years), while previous studies analyzed isolates belonging to a single hospital location and collected less than 1 year apart. Secondly, we combined cgMLST and cgSNP phylogenetic analysis in order to improve the resolution of the WGS-based reference method, as previously described (Raro et al., 2020). Such combination allowed the identification of the best allele and SNP cutoffs for cluster definition of our WGS data. It is important to note that a universal genomic cutoff value to determine whether different isolates belong to an outbreak does not currently exist (López et al., 2021). Less than 15 alleles of distance in pairwise comparisons between isolates has been suggested for outbreak delimitation by cgMLST (Lepuschitz et al., 2019; Miro et al., 2020). However, this cutoff may not always be reliable as genetic variability depends on the time elapsed among the collection dates of isolates. Similarly, if non-identical sequences share a monophyletic origin, the identification of the isolates that belong to an outbreak may be difficult, as a defined SNP cutoff has not yet been established. It should also be pointed out that each laboratory must internally validate its FTIR cutoff for clustering analysis with a reference method, given that the manufacturer has an unofficial range of cutoff values for *K. pneumoniae* (0.20–0.25) based on information gathered from internal validations of different laboratories (Rakovitsky et al., 2020). The cutoff applied in the present study after internal validation (0.240) was similar to those used by Hu et al. (0.221) and Martak et al. (0.220; Martak et al., 2019; Hu et al., 2021).

Genome composition of *K. pneumoniae* clones may slightly vary over time and from patient to patient, generating phenotypic differences that are not detected by FTIR. As recommended by the manufacturer, only the default wavelength region, which comprises the polysaccharide region, was analyzed by FTIR in the present study; thus, other existing differences/similarities at the level of proteins or fatty acids could not be detected. Nevertheless, and despite the expected lower discriminatory power of FTIR for clustering identification than WGS, when the clustering performances of CE and FTIR were compared, both AR and AW were significantly higher for FTIR clustering than CE clustering. Accordingly, FTIR inferred more true genomic relationships than epidemiological criteria, while a similar proportion of genomic singletons was detected by both FTIR and epidemiological criteria. In an epidemiologically suspected outbreak scenario, a rapid result is required to confirm whether isolates are closely related or not. The detection of related isolates involves the implementation of the most appropriate infection control measures as soon as possible. Therefore, although FTIR clustered 38.1% of genomic singletons, in the clinical practice, misdiagnosis of singletons as related isolates by FTIR clustering might be desirable over the opposite because it promotes the implementation of outbreak control measures to limit the potential spread of the outbreak, while confirmation by WGS is being performed. Contrarily to WGS, the IR Biotyper is a rapid (3–4 h), low-cost method with a simple sample preparation that can be easily implemented in the everyday routine of a microbiology laboratory after the overnight culture of the isolate of interest.

Importantly, FTIR was able to detect a relationship for isolates belonging to four clusters hidden to the CE investigations because the isolates were obtained from different hospital wards/units and/or differed in collection date by more than one month, evidencing the difficulty to establish an epidemiological link among them. Thus, we suggest that a prospective surveillance approach should be carried out in order to identify all potential hidden epidemiological clusters of isolates from patients or environmental reservoirs; this could be easily implemented using FTIR as a first-line surveillance tool, while WGS could be performed for clustering confirmation as well as for genomic characterization of circulating isolates at the level of ST, resistome, and virulome. As other studies describe, high-risk clones ST147, ST307, and ST392 are frequently associated with ESBL production and detected in healthcare settings in Spain (D’Apolito et al., 2020; Peirano et al., 2020). Particularly, in the present study these high-risk clones represented more than 60% of studied isolates, being ST307 the most prevalent one (34.9%). It is also important to remark that previous studies have demonstrated that outbreak delimitation based on ST determination might not be adequate (Zhou et al., 2017; Gona et al., 2020). In this sense, the detection of different genomic clusters within ST307 in our geographical region (Figure 2A) probably indicates multiple introductions of ST307 into the hospital setting.

The use of the FTIR in the clinical microbiology practice has potential limitations. Firstly, FTIR spectra analysis does not provide species identification. In a hypothetical situation of an outbreak, an accidentally mixed culture for any of the isolates could potentially lead to its misclassification as a non-related strain. This limitation can be easily resolved by confirming species identification using MALDI-TOF MS (or other bacterial identification techniques). Secondly, the FTIR technique requires a standardization of the culture medium and incubation times of the samples (Dinkelacker et al., 2018; Quintelas et al., 2018; Hu et al., 2021); the use of different medium or incubation times may affect the growth and the cell-wall composition and, therefore, the spectra quality and subsequent analysis.

Our study also has several limitations. Firstly, not all ESBL-Kp isolates detected in the hospital over the study period could be included in this study, concealing the detection of any further potential outbreak relationships or the detection of environmental reservoirs. Secondly, only one biological replicate from each isolate was analyzed; thus, the inherent biological variability could not be taken into account. Finally, although the FTIR operated under standard laboratory conditions, temperature and hygrometry were not controlled. Since FTIR spectroscopy techniques are known to be very sensible to variations in these two physical properties, it is not known to what extent their fluctuations might have affected the results of this study.

## Conclusion

FTIR is a rapid, low-cost, and easy-to-use phenotypic method that could be used in the clinical microbiology laboratory as first-line tool for the rapid identification of ESBL-Kp outbreaks, while the WGS analyses are being performed for confirmation and further characterization. Thus, integrating FTIR results into epidemiological investigations may add valuable information for infection control in the hospital setting.

## Data Availability Statement

The data for this study have been deposited in the European Nucleotide Archive (ENA) at EMBL-EBI under accession number PRJEB51676.

## Ethics Statement

Ethical review and approval was not required for the study on human participants in accordance with the local legislation and institutional requirements. Written informed consent for participation was not required for this study in accordance with the national legislation and the institutional requirements.

## Author Contributions

P-JC conceived the study. EM, VS, MG, and P-JC designed the study. LC, NS, IC, and MG performed the epidemiological analyses. JW-W, MQ, AT, and MN performed the routine microbiological analyses. JW-W, AT, and MN performed the FTIR experiments. JW-W and VS performed the WGS experiments. JW-W, AB, MP-V, MG-M, and VS performed the bioinformatics analyses. JW-W, AB, and VS analyzed and interpreted the data and wrote the first draft of the manuscript. AB created the figures and performed the statistical analyses. JW-W, AB, EM, MP-V, VS, MG, and P-JC reviewed and edited the manuscript. All authors contributed to the article and approved the submitted version.

## Conflict of Interest

The authors declare that the research was conducted in the absence of any commercial or financial relationships that could be construed as a potential conflict of interest.

## Publisher’s Note

All claims expressed in this article are solely those of the authors and do not necessarily represent those of their affiliated organizations, or those of the publisher, the editors and the reviewers. Any product that may be evaluated in this article, or claim that may be made by its manufacturer, is not guaranteed or endorsed by the publisher.
