# Supplementary material for: Evaluation of Fourier Transform Infrared Spectroscopy as a First-Line Typing Tool for the Identification of Extended-Spectrum β-Lactamase-Producing Klebsiella pneumoniae Outbreaks in the Hospital Setting
Source: Front Microbiol. 2022 Jun 9;13:897161. doi: 10.3389/fmicb.2022.897161 (PMC9218594; doi:10.3389/fmicb.2022.897161)
Supplement: Supplementary file 1 [file Data_Sheet_1.docx]

### *Supplementary Material*

# Supplementary Text 1

***Results: Distribution of isolates according to source and detection ward***

Over the study period, sixty-three isolates of ESBL-Kp were included from 61 different hospitalized patients and two environmental sites. The isolates were detected in the intensive care unit (ICU, *n*=21), the nephrology department (NPHD, *n*=12), two different SARS-CoV-2 intensive care units (SICU 1 and SICU 2, *n*=10), the geriatric department (GD, *n*=7), the cardiac care unit (CCU, *n*=5), the obstetric ward (OW, *n=*2), the cardiology department (CD, *n*=1), the infectious disease department (IDD, *n*=1), the neurology department (ND, *n*=1), the neonatal intensive care unit (NICU, *n*=1), the oncohematology department (OHD, *n*=1), and the surgery department (SD, *n*=1).

Patient isolates sources were rectal swab (*n*=44), urine (*n*=12), sputum (*n*=3), wound exudate (*n*=1), and blood culture (*n*=1). Environmental isolates (*n*=2) were collected from a sink at the ICU and from a mattress at the SD **(Supplementary Table 1)**.

# Supplementary Text 2

***Results: Mean allele distances between isolates of a genomic cluster***

According to whole-genome sequencing (WGS) results, 11 different clusters were detected. The mean allele distances between isolates from a genomic cluster ranged from 1 to 9.5. Specifically, six genomic clusters (III-VIII) included two isolates; the allele distances were 2, 10, 7, 1, 3, and 15 between isolates of clusters III, IV, V, VI, VII, and VIII, respectively. Additionally, five clusters (I, II, IX, X, and XI) comprised more than two isolates and presented an average allele distance of 9.5 (range, 0-18; *n*=16), 3.2 (range, 0-6; *n*=4), 2 (range, 1-3; *n*=3), 0.5 (range, 0-1; *n*=4), and 7 (range, 11-17; *n*=3), respectively.

# Supplementary Text 3

***Results: Description of FTIR clusters that affects*** ***more than one hospital location over the study period***

As WGS, FTIR also detected clusters in more than one hospital location: (i) cluster 1 isolates were detected in the CCU, NPHD, ICU, and SICU 1; (ii) cluster 2 isolates were present in the CCU, ND and SD; (iii) cluster 4 isolates were present in the ICU, NPHD, OHD, and SICU 1; (iv) cluster 5 isolates were detected in the ICU and CCU; (v) cluster 6 isolates were detected in the SICU 1 and CD; (vi) cluster 10 isolates were detected in the GD and ICU (**Supplementary Table 1**). However, four (1, 4, 6, and 10) out of these six clusters were not fully concordant with WGS clustering (**Table 1**). Cluster 1 included only 13 out of the 16 isolates that formed genomic cluster I. Cluster 4 grouped together the two isolates of genomic cluster IV, however, FTIR also grouped within cluster 4 five genomic singletons and nine additional isolates of different genomic clusters. Cluster 6 included two out of the three isolates forming genomic cluster XI. Finally, cluster 10 grouped together two genomic singletons. Conversely, clusters 2 and 5 perfectly matched clusters II and V, respectively.


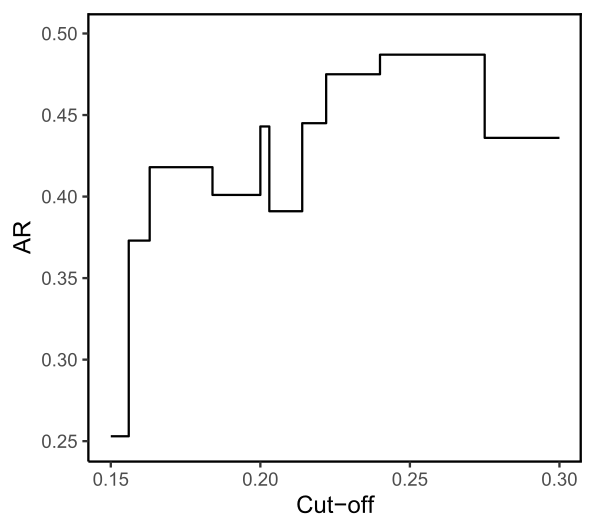


**Supplementary Figure 1.** Final internal validation of FTIR clustering cut-off value. The most restrictive cut-off value that maximized the Adjusted Rand index (AR) was 0.240 for the clustering of the study dataset (N=63) of extended-spectrum β-lactamase-producing *Klebsiella pneumoniae* isolates considering WGS as the reference method. The tested cut-off range corresponds to the unofficial range suggested by the manufacturer (0.20-0.25) with an additional 0.05 range on each side (0.15-0.30).

**Supplementary Table 1.** **Comparison of the three clustering methods for each ESBL-Kp isolate.**

| Isolate ID | Collection date | Isolate source | Hospital location | ST | Epidemiological cluster | FTIR cluster | WGS cluster |
| --- | --- | --- | --- | --- | --- | --- | --- |
| 1 | 07/16/2018 | RS | CCU | 392 | A | 1 | I |
| 5 | 07/23/2018 | RS | CCU | 392 | A | Singleton | I |
| 13 | 08/02/2019 | U | NPHD | 392 | Singleton | 1 | I |
| 14 | 08/07/2019 | U | NPHD | 392 | C | 1 | I |
| 15 | 08/11/2019 | RS | NPHD | 392 | C | 1 | I |
| 16 | 08/21/2019 | RS | NPHD | 392 | C | 1 | I |
| 21 | 08/31/2020 | RS | ICU | 392 | D | 1 | I |
| 22 | 10/05/2020 | U | NPHD | 392 | Singleton | 1 | I |
| 31 | 02/15/2021 | RS | SICU 1 | 392 | F | 1 | I |
| 33 | 02/22/2021 | RS | SICU 1 | 392 | F | 1 | I |
| 35 | 03/03/2021 | RS | OHD | 392 | Singleton | 4 | I |
| 37 | 03/10/2021 | U | NPHD | 392 | Singleton | 1 | I |
| 41 | 03/29/2021 | RS | SICU 2 | 392 | G | Singleton | I |
| 43 | 04/13/2021 | RS | ICU | 392 | E | 1 | I |
| 57 | 05/17/2021 | RS | NPHD | 392 | H | 1 | I |
| 58 | 05/17/2021 | RS | NPHD | 392 | H | 1 | I |
| 2 | 07/18/2018 | RS | CCU | 307 | A | 2 | II |
| 3 | 07/21/2018 | U | CCU | 307 | A | 2 | II |
| 4 | 07/23/2018 | RS | ND | 307 | Singleton | 2 | II |
| 62 | 10/11/2021 | EI | SD | 307 | Singleton | 2 | II |
| 6 | 08/16/2018 | U | OW | 147 | B | 3 | III |
| 7 | 08/17/2018 | U | OW | 147 | B | 3 | III |
| 17 | 07/20/2020 | RS | ICU | 307 | D | 4 | IV |
| 59 | 05/25/2021 | RS | NPHD | 307 | H | 4 | IV |
| 20 | 08/24/2020 | RS | ICU | 45 | Singleton | 5 | V |
| 40 | 03/25/2021 | U | CCU | 45 | Singleton | 5 | V |
| 23 | 10/08/2020 | BC | ICU | 307 | D | 4 | VI |
| 24 | 10/13/2020 | RS | ICU | 307 | D | 4 | VI |
| 26 | 10/28/2020 | RS | ICU | 307 | D | 4 | VII |
| 27 | 11/30/2020 | RS | ICU | 307 | D | 4 | VII |
| 30 | 02/15/2021 | RS | SICU 1 | 307 | F | 4 | VIII |
| 61 | 10/07/2021 | S | GD | 307 | Singleton | 4 | VIII |
| 42 | 04/13/2021 | RS | ICU | 307 | E | 7 | IX |
| 47 | 04/26/2021 | RS | ICU | 307 | E | 7 | IX |
| 52 | 05/04/2021 | RS | ICU | 307 | E | 7 | IX |
| 44 | 04/19/2021 | RS | SICU 2 | 2703 | G | 8 | X |
| 49 | 04/27/2021 | RS | SICU 2 | 2703 | G | 8 | X |
| 53 | 05/10/2021 | RS | SICU 2 | 2703 | G | 9 | X |
| 56 | 05/12/2021 | U | SICU 2 | 2703 | G | 9 | X |
| 32 | 02/19/2021 | RS | SICU 1 | 15 | F | 6 | XI |
| 34 | 02/26/2021 | RS | CD | 15 | Singleton | 6 | XI |
| 45 | 04/20/2021 | U | GD | 15 | Singleton | Singleton | XI |
| 8 | 05/16/2019 | RS | NICU | 628 | Singleton | Singleton | Singleton |
| 9 | 07/25/2019 | S | GD | 336 | Singleton | Singleton | Singleton |
| 10 | 07/29/2019 | RS | GD | 17 | Singleton | Singleton | Singleton |
| 11 | 07/29/2019 | RS | GD | Unknown | Singleton | Singleton | Singleton |
| 12 | 07/31/2019 | U | NPHD | 307 | Singleton | 4 | Singleton |
| 18 | 07/27/2020 | RS | ICU | 485 | D | Singleton | Singleton |
| 19 | 08/17/2020 | RS | ICU | 307 | D | 4 | Singleton |
| 25 | 10/13/2020 | RS | ICU | 54 | D | Singleton | Singleton |
| 28 | 12/07/2020 | RS | ICU | 147 | E | 3 | Singleton |
| 29 | 12/21/2020 | RS | ICU | 307 | E | 4 | Singleton |
| 36 | 03/10/2021 | RS | ICU | 307 | Singleton | 4 | Singleton |
| 38 | 03/16/2021 | RS | ICU | 307 | E | 4 | Singleton |
| 39 | 03/22/2021 | RS | GD | 792 | Singleton | Singleton | Singleton |
| 46 | 04/26/2021 | S | IDD | 15 | Singleton | Singleton | Singleton |
| 48 | 04/27/2021 | WE | NPHD | 268 | Singleton | Singleton | Singleton |
| 50 | 04/27/2021 | RS | ICU | 29 | Singleton | Singleton | Singleton |
| 51 | 05/04/2021 | RS | ICU | 14 | E | Singleton | Singleton |
| 54 | 05/10/2021 | U | NPHD | 391 | H | Singleton | Singleton |
| 55 | 05/10/2021 | RS | SICU 2 | 556 | G | Singleton | Singleton |
| 60 | 07/02/2021 | EI | ICU | 307 | Singleton | 10 | Singleton |
| 63 | 10/11/2021 | RS | GD | 307 | Singleton | 10 | Singleton |

BC: Blood culture, CCU: Cardiac care unit, CD: Cardiology department, EI: Environmental isolate, GD: Geriatric department, ICU: Intensive care unit, IDD: Infectious disease department, ND: Neurology department, NICU: Neonatal intensive care unit, NPHD: Nephrology department, OHD: Oncohematology department, OW: obstetric ward, RS: Rectal swab, S: Sputum, SD: Surgery department, SICU 1: SARS-CoV-2 intensive care unit 1, SICU 2: SARS-CoV-2 intensive care unit 2, ST: Sequence type, U: Urine, WE: Wound exudate.
